# A Novel 8-Gene Prognostic Signature for Survival Prediction of Uveal Melanoma

**DOI:** 10.1155/2021/6693219

**Published:** 2021-08-14

**Authors:** Zhongjun Tang, Kebo Cai

**Affiliations:** ^1^Department of Ophthalmology, Minhang Hospital, Fudan University, Shanghai, China; ^2^Department of Ophthalmology, Xinhua Hospital Affiliated to Shanghai Jiao Tong University School of Medicine, Shanghai, China

## Abstract

**Background:**

Uveal melanoma (UM) has favorable local tumor control, but once metastasis develops, the prognosis is rather poor. Thus, it is urgent to develop metastasis predicting markers.

**Objective:**

Our study investigated a novel gene expression-based signature in predicting metastasis for patients with UM.

**Methods:**

In the discovery phase, 63 patients with UM from GEO data set GSE22138 were analyzed using the Weighted Correlation Network Analysis (WGCNA) to identify metastasis-related hub genes. The Least Absolute Shrinkage and Selection Operator (Lasso) Cox regression was used to select candidate genes and build a gene expression signature. In the validation phase, the signature was validated in The Cancer Genome Atlas database.

**Results:**

Forty-one genes were identified as hub genes of metastasis by WGCNA. After the Lasso Cox regression analysis, eight genes including RPL10A, EIF1B, TIPARP, RPL15, SLC25A38, PHLDA1, TFDP2, and MEGF10 were highlighted as candidate predictors. The gene expression signature for UM (UMPS) could independently predict MFS by univariate and multivariate Cox regression analysis. Incorporating UMPS increased the AUC of the traditional clinical model. In the validation cohort, UMPS performed well in predicting the MFS of UM patients.

**Conclusions:**

UMPS, an eight-gene-based signature, is useful in predicting prognosis for patients with UM.

## 1. Introduction

Uveal melanoma (UM), arising from the melanocytes resident of the eye, is the most common type of primary intraocular malignant tumor in adults [1]. It occurs in approximately 1 per 200,000 Americans annually [2]. Despite favorable local tumor control by means of radiotherapy, UM is a highly aggressive disease with moderate metastatic risk due to micrometastasis before treatment [1]. Upon the diagnosis of metastasis, the patients' survival time was less than 12 months [3]. Several clinical trials were designed to evaluate adjuvant therapy for patients with a high risk of metastasis to improve disease outcome [3]. Identifying patients that are prone to have disease progression via postoperative metastasis risk stratification may help with decision-making of strategies for the surveillance and adjuvant therapy.

Accumulating evidence suggested that many clinicopathological or genomic predictors of metastatic disease may be useful for patients' risk stratification. Several studies found that large tumor size, extraocular extension, male gender, and an epithelioid cell type can predict high metastatic risk [3]. Molecular investigation of UM could provide information for disease progression and metastasis. Previous studies have highlighted several promising genetic biomarkers. However, few biomarkers have been incorporated into clinical practice. The limitations of these researches included a single-center cohort and a limited methodology for feature selection.

To best identify patients at high risk of metastasis, we conducted the Weighted Correlation Network Analysis (WGCNA) and Least Absolute Shrinkage and Selection Operator (Lasso) Cox regression in a gene expression microarray data to selected candidate genes and trained a novel eight-gene expression signature. Our findings were externally validated in The Cancer Genome Atlas (TGCA) dataset.

## 2. Materials and Methods

### 2.1. Patients and Design

**Figure 1:**
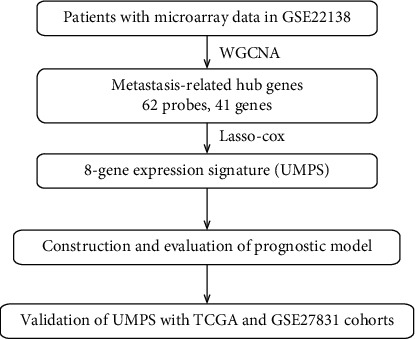
Flow chart of study design.

The training cohort GSE22138 contains 63 tumor samples from UM patients that was downloaded from the GENE EXPRESSION OMNIBUS (GEO) database. Gene expression was analyzed on GeneChip Human Genome U133 Plus 2.0 microarrays (Affymetrix). The TCGA cohort consists of 80 tumor tissue samples from UM patients. Level 3 RNA sequencing data was downloaded via UCSC Xena browser (https://xenabrowser.net). The GSE27831 cohort contains 29 tumor tissue samples from UM patients. The study design was illustrated in Figure 1.

### 2.2. Identifying Metastasis Related Hub Genes

The coexpression modules for microarray data were developed using the WGCNA method [4]. In our study, the soft-thresholding was set as 10 and minimum gene module size as 30. Modules with absolute correlation coefficients values between metastasis status more than 0.15 were identified as metastasis-related modules. The module with the highest correlation coefficients was subject to further downstream feature selection. Hub genes underwent gene ontology (GO) function and KEGG pathway enrichment analyses using “clusterProfiler” R package.

### 2.3. Feature Selection and Signature Construction

To select metastasis-related features, we considered three grouped variable selection methods. The ridge regression (alpha = 0) shrinks coefficients of correlated predictors, while Lasso regression (alpha = 1) tends to choose one of them and discard the other features (shrinks all the way to zero). Although ridge regression reduces the complexity of the model, it does not really solve the problem of feature selection. The elastic network compromises between the ridge regression and Lasso penalties (alpha is from 0 to 1). While setting alpha from 0 to 1, the cv error is minimized for alpha = 1. Based on this, we choose Lasso regression. Finally, to consider the issue of survival time, the Least Absolute Shrinkage and Selection Operator (Lasso) Cox regression method [5] was then applied from the primary cohort. The penalty parameter tuning was conducted by 10-fold cross-validation. In this method, weak coefficients of predicting features shrink to zero and the strongest prognostic features remained. A signature formula (uveal melanoma metastasis prediction score, UMPS) to predict the metastasis-free survival of UM patients was generated by the linear combination of the final selected gene expression features weighted by their individual coefficients from the Lasso Cox regression analysis.

### 2.4. Functional Annotation of the Risk Score

Gene set enrichment analysis (GSEA) [3] was done by GSEA 4.1 command line version on the Linux system. Spearman correlation coefficients of the risk score and each gene were input as the prerank gene list. The “h.all.v7.1.symbols.gmt” gene sets were set as the gene sets for enrichment analysis. The immune cell infiltration status was evaluated by the CIBERSORT software (https://cibersort.stanford.edu/) [3] with the LM22 (22 immune cell types) as the signature gene file. The analysis was performed with a 1,000-time permutation.

### 2.5. Evaluation and Clinical Use of Signature

Survival curves of different risk groups were plotted by the Kaplan-Meier (K-M) method. The between-group survival difference was tested by the log-rank test (*p* < 0.05). Univariate and multivariate analyses with Cox proportional hazards regression were applied with the determined predictors of survival. Time-dependent receiver operating characteristic (ROC) curve analysis was applied to calculate the Area Under Curve (AUC) of different prediction models. The clinicopathological factors with *p* < 0.05 by univariate Cox regression were merged as the Clinical model. Decision curve analysis was applied to calculate the net benefit from the use of the signature model, the American Joint Committee on Cancer (AJCC) Tumor-Node-Metastasis (TNM) stage, and the combined signature and AJCC TNM stage model at different threshold probabilities [3].

### 2.6. Statistical Analysis

All statistical analysis was performed using the R software (version 3.6.2, R Project for Statistical Computing, http://www.r-project.org). WGCNA was performed with the “WGCNA” package. Immune cell proportions between low risk and high risk were evaluated by the Wilcoxon test. Cox proportional hazards regression was conducted with the “survival” package. Time-dependent ROC with AUC analysis was conducted with the “survivalROC” package. Lasso Cox regression analysis was conducted with the “glmnet” package. Decision curve analysis was performed with the “dca” package. Statistical tests with a two-sided *p* value less than 0.05 was considered significant.

## 3. Results

### 3.1. Identification of Metastasis Associated Modules by WGCNA

**Figure 2:**
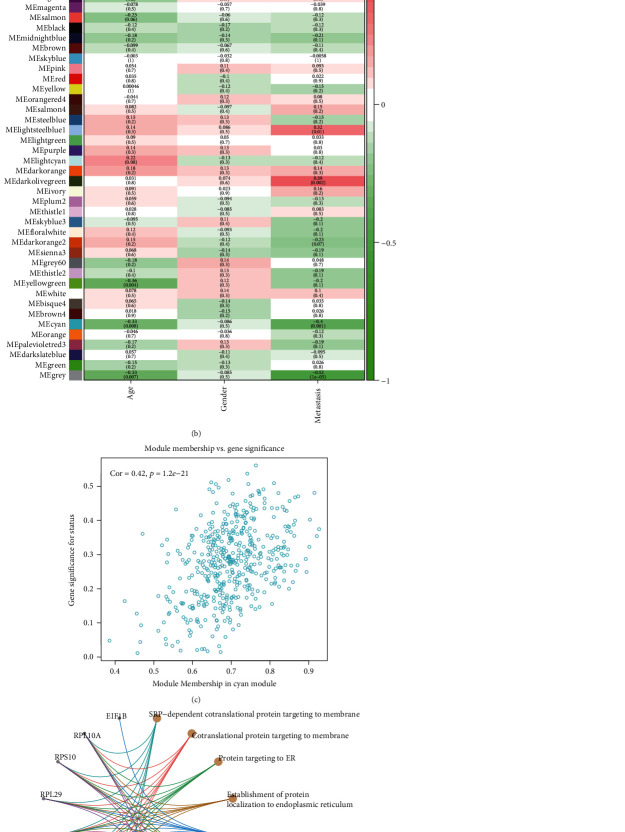
WGCNA of metastasis-related module. (a) The clustering dendrograms showed 54 coexpression modules recognized by WGCNA. (b) The correlations and *p* value of module-traits associations. (c) The cyan module genes were highly correlated with metastasis status. (d) Gene Ontology (GO) analyses of the 41 hub genes. (e) KEGG pathways analysis of the 41 hub genes.

To construct gene coexpression modules, microarray data from GSE22138 was subjected to WGCNA. After setting the power as 10, 54 coexpression modules were constructed and assigned with different colors (Figure 2(a)). The relationships between metastasis status and the identified gene modules are presented in Figure 2(b). Among the modules, module “blue”, “cyan”, “darkgrey”, “darkolivegreen”, “lightsteelblue1”, “tan”, and “grey” were the most relevant modules with metastasis status (Figure 2(b)). The genes in the cyan module turned out to be highly correlated with metastasis (Figure 2(c)). With a cutoff of correlation ≥0.8, 62 hub probes (41 hub genes) from the cyan module were chosen for further analysis. GO analyses of the 41 hub genes suggested the biological processes enriched were SRP-dependent cotranslational protein targeting to membrane, cotranslational protein targeting to membrane, and protein targeting to ER et al. (Figure 2(d)). Enrichment KEGG pathways indicated that the Ribosome pathway was enriched (Figure 2(e)).

### 3.2. Constructing an Eight-Gene Metastasis-Free Survival Prediction Signature

**Figure 3:**
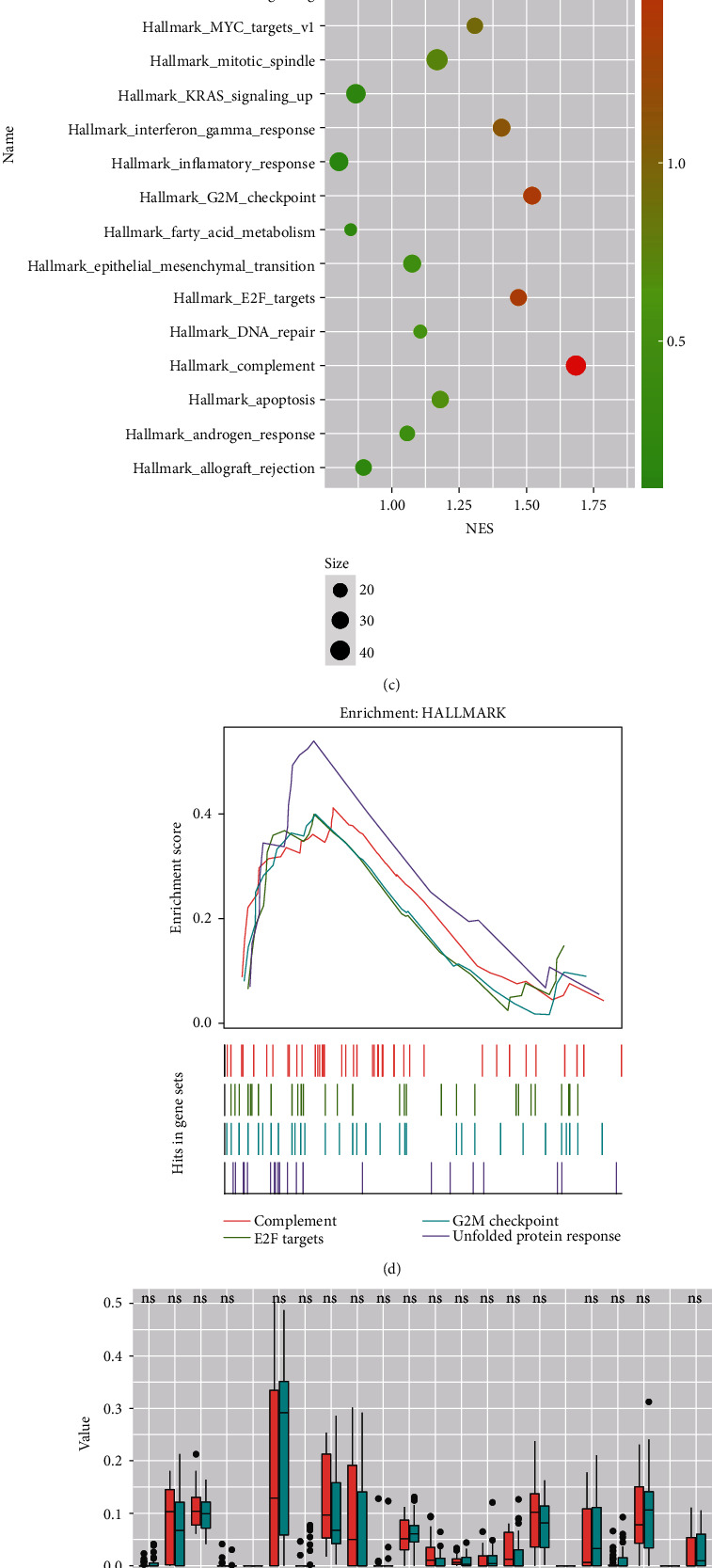
Construction of the 8 gene-based signature. (a) Cross-validation for tuning parameter selection in the Lasso Cox model. (b) Individual coefficient of each of the 8 markers. (c, d) GSEA analysis according to “hallmark gene sets.” The complement, E2F targets, G2M checkpoint, and unfolded protein response pathways were activated in the high-risk group (*p* < 0.05). (e, f) CIBERSORT showed no difference in immune cell fractions in the low-risk and high-risk groups.

Then, Lasso Cox regression analysis was performed to identify the most predictive markers among the 41 genes. The ten-fold cross-validation for tuning parameter selection was shown in Figure 3(a). Then, eight genes were selected as the predicting feature. The final genes were RPL10A, EIF1B, TIPARP, RPL15, SLC25A38, PHLDA1, TFDP2, and MEGF10. The individual coefficient of each of the eight genes by Lasso Cox analysis suggested that seven genes were protective (Hazard Ratio, HR <1) and one gene was associated with high risk (HR >1) (Figure 3(b)). According to the coefficient weighed by Lasso Cox regression analysis, the UMPS was calculated as follows: UMPS = (0.720∗expression level of RPL10A) + (−0.354∗expression level of EIF1B) + (−0.076∗expression level of TIPARP) + (−0.434∗expression level of RPL15) + (−0.066∗expression level of SLC25A38) + (−0.044∗expression level of PHLDA1) + (−0.397∗expression level of TFDP2) + (−0.017∗expression level of MEGF10). GSEA analysis according to the gene list correlated with the risk score showed that the high-risk group was associated with the complement, E2F targets, G2M checkpoint, and unfolded protein response pathways (*p* < 0.05) (Figures 3(c) and 3(d)). There was no difference in the immune cell proportions between the low-risk and high-risk groups (Figures 3(e) and 3(f)), indicating that high-UMPS patients' worse prognosis may not be due to differed immune cell infiltration in the tumor microenvironment.

### 3.3. Survival Prediction Based on Risk Score of the UMPS in the Training Cohort

**Figure 4:**
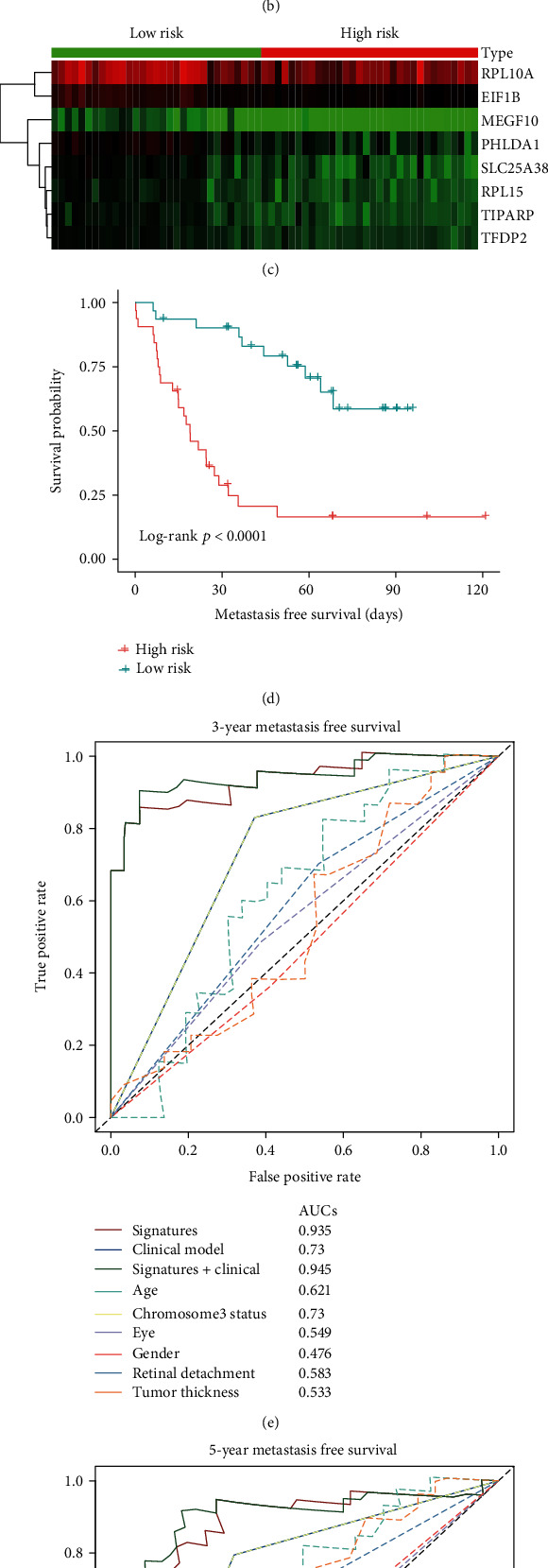
Survival analysis, prognostic performance, and clinical utility of UMPS in the training cohort. (a) Risk score distribution of the training cohort. (b) Survival overview. (c) Heatmap showing the expression of the 8 genes in the low-risk and high-risk groups. (d) Survival curve of the low-risk and high-risk groups by Kaplan-Meier survival analysis. The high-risk group had worse metastasis-free survival than the low-risk group. (e, f) Time-dependent receiver operating characteristic (ROC) analysis for comparing the performance of UMPS with the clinicopathological factors. The AUC of UMPS was higher than the single clinicopathological factor in prediction 3-y and 5-y metastasis-free survival.

Then, we investigate the association between the UMPS and metastasis-free survival in the training cohort. According to the UMPS formula, the risk score for each patient was calculated. Patients were divided by the median level of risk scores into a low-risk group (*n* = 31) and a high-risk group (*n* = 32) (Figure 4(a)). The survival time distributions were illustrated in Figure 4(b). The expression heatmap of the genes in the formula showed that patients in the high-risk group had relatively lower expression of EIF1B, TIPARP, RPL15, SLC25A38, PHLDA1, TFDP2, and MEGF10 and higher expression of RPL10A (Figure 4(c)). Patients in the high-risk group had significantly worse metastasis-free survival compared to those in the low-risk group as shown in the Kaplan-Meier survival curve (*p* < 0.001) (Figure 4(d)).

### 3.4. Prognostic Performance and Clinical Utility of UMPS in the Training Cohort

First, univariate and multivariate Cox proportional hazards regression analysis were performed. Samples that had covariates with Not Applicable (N/A) values were excluded. The tests of proportional hazards assumption in the remaining 51 patients indicate that Chromosome3 status (HR = 4; *p* < 0.001) and UMPS (HR = 6.2; *p* < 0.001) were associated with metastasis-free survival (MFS). In the multivariate Cox Regression model, UMPS (HR = 6.09; *p* < 0.05) was found to be independently associated with metastasis-free survival (supplementary table 1**)**. Second, time-dependent ROC analysis showed the AUC was 0.935 and 0.893 for UMPS in predicting 3-y and 5-y metastasis-free survival, respectively (Figures 4(e) and 4(f)). AUC of UMPS was higher than any single clinicopathological predictor including Chromosome3 status (AUC = 0.730 and 0.738 for 3-y and 5-y, respectively) (Figures 4(e) and 4(f)). According to the univariate Cox regression model, the Chromosome3 status was incorporated into the Clinical Model in the training cohort. The combination of UMPS and Clinical model (AUC = 0.945 and 0.901 for 3-y and 5-y, respectively) could improve the accuracy of the clinical model (Figures 4(e) and 4(f)).

### 3.5. Validation of the UMPS for Survival Prediction in Additional Cohorts

**Figure 5:**
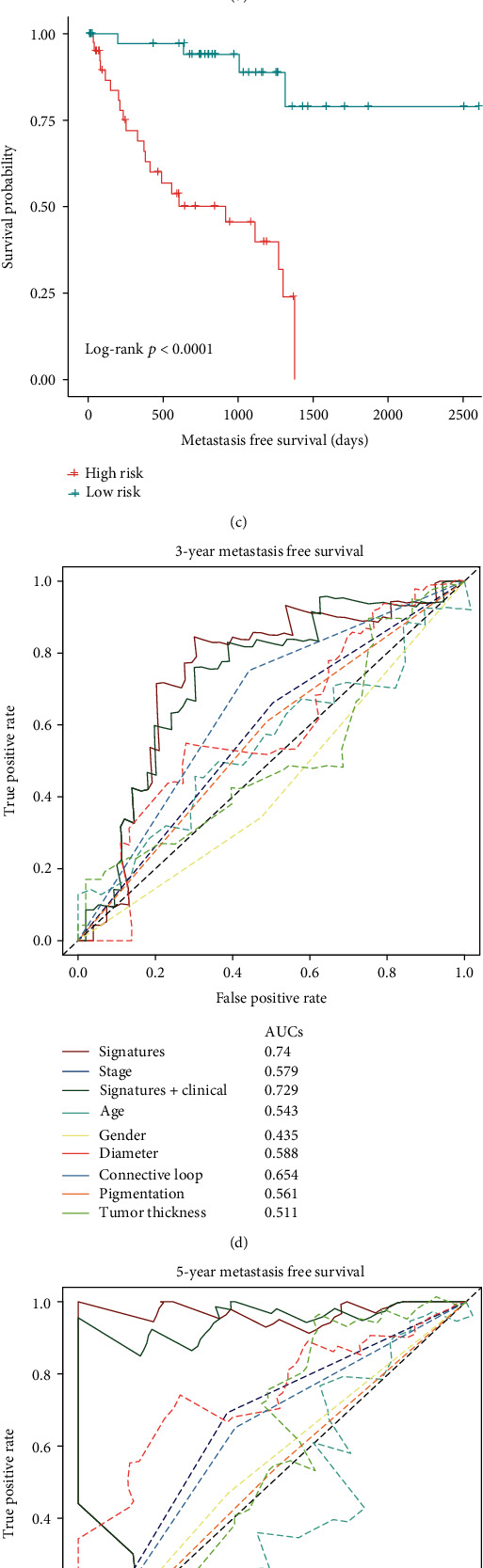
Validation of UMPS in the TCGA cohort. (a) Risk score distribution. (b) Survival overview. (c) Survival curve of UMPS for metastasis-free survival. The high-risk group had worse metastasis-free survival than the low-risk group. (d, e) Time-dependent receiver operating characteristic (ROC) analysis for comparing the performance of UMPS and the clinicopathological factors. The AUC of UMPS was higher than the single clinicopathological factor in predicting 3-y and 5-y metastasis-free survival. The addition of UMPS could improve the performance of the AJCC TNM staging system.

At the validation phase, we validated the prognostic performance of the UMPS in the TCGA cohort and GSE27831 cohort. Using the UMPS formula derived from the training set, we calculated the risk score for each patient. Risk score distribution and survival overview were shown in Figures 5(a) and 5(b), respectively. According to the median cut-off value, patients were separated into low-risk (*n* = 40) and high-risk (*n* = 40) groups. High-risk score of UMPS was associated with worse MFS, disease-specific survival (DSS), and overall survival (OS) in the TCGA cohort as shown in the Kaplan-Meier survival curve (all *p* < 0.001) (Figure 5(c), Supplementary Fig. 1). In addition, UMPS could predict disease-free survival (DFS) in the GSE27831 cohort (*p* = 0.017, Supplementary Fig. 2a-c).

### 3.6. Prognostic Power for UMPS in the Additional Cohorts

**Figure 6:**
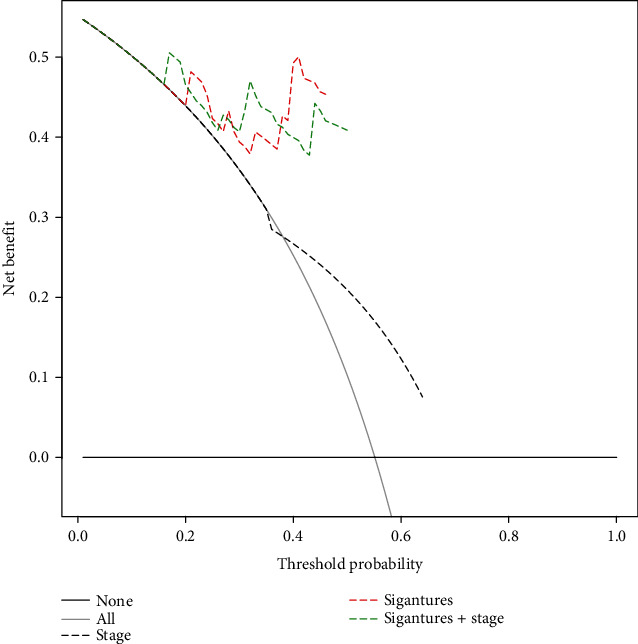
Decision curve analysis for each model in the validation cohort. The *y*-axis measures the net benefit. The net benefit was calculated by summing the benefits (true-positive results) and subtracting the harms (false-positive results), weighting the latter by a factor related to the relative harm of an undetected metastasis compared with the harm of unnecessary treatment. The UMPS or the combined UMPS and AJCC TNM staging model had the higher net benefit compared with the AJCC TNM staging system in 10% threshold probabilities.

Seventy-four samples that had non-N/A covariates were included for prognostic power evaluation. Similarly, high UMPS was an independent risk factor for MFS, DSS, and OS evaluated by univariate and multivariate Cox Regression Analysis (supplementary table 2). Time-dependent ROC analysis suggested that UMPS was more accurate in predicting 3-y and 5-y MFS (AUC = 0.740 and 0.951, respectively) compared to any clinicopathological parameter alone, including AJCC TNM stage (AUC = 0.579 and 0.654, respectively) (Figures 5(d) and 5(e)). The AJCC TNM stage is the current gold standard for the prognostic stratification of UVM. Thus, it was combined with UMPS for time-dependent ROC analysis, despite that it was not associated with MFS in univariate Cox Regression analysis (*p* = 0.059). The addition of UMPS could improve predicting accuracy of the traditional AJCC TNM stage model. Finally, decision curve analysis demonstrated that the UMPS or the combination of UMPS and AJCC TNM staging outperformed the traditional AJCC TNM staging system in terms of clinical usefulness for predicting 5-y MFS (Figure 6). Additionally, time-dependent ROC analysis showed that UMPS was more accurate in predicting 3-y and 5-y DFS (AUC = 0.794 and 0.867, respectively) compared to any clinicopathological parameter alone in the GSE27831 cohort (Supplementary Fig. 2d-e).

## 4. Discussion

One challenge in UM management is that, despite favorable local tumor control of various treatment options, such as stereotactic radiotherapy, enucleation, brachytherapy, and proton therapy, patients who progressed to metastatic disease have poor prognosis [6]. In this study, we used WGCNA to screen hub genes correlated to metastasis in a discovery cohort from the GEO database and selected 8 significant metastasis-associated genes by using Lasso Cox regression analysis. GSEA analysis indicated that various metastasis stimulating pathways were activated in the high-risk group. Subsequently, we built a gene expression signature according to the 8 identified genes named UMPS in the discovery cohort which was an independent factor to predict metastasis-free survival by multivariate Cox regression model. UMPS had superior accuracy than the clinical model in predicting 3-y and 5-y MFS. The addition of UMPS could improve the performance of the traditional clinical predicting model. Finally, we validated the UMPS in the TCGA cohort. Our results suggested that UMPS could be helpful in stratifying UM patients into distinct subgroups with different risks of metastasis. The UMPS could simplify the decision-making process for patients with UM in terms of individualized surveillance and therapeutic strategies.

Our study represents a robustly discovered and validated gene expression signature with an orientation to predict metastasis as a complement to the traditional clinicopathological parameter-based approaches. So far, various clinicopathological prognostic predictors of UM had been widely researched. The AJCC TNM staging is a well-established approach categorizing UM patients in terms of predicting prognosis [3]. In the study by Shields et al., the reported metastasis rates at ten years were 12% for T1, 29% for T2, and 61% for T3 [7]. Recent studies found that older age is associated with an unfavorable prognosis [8]. By the three age characterization strategy (≤20 years, young; 21-60 years, midadults; >60 years, older adults), Kaliki et al. found that younger patient of age is associated with lower incsdence of metastasis compared with the other two groups [8]. Besides, investigations also found that the tumor size is a critical factors to predict the metastasis and prognosis for patients with UM [3]. For UM patients with small tumors (<10 mm), medium tumors (10-15), and large tumors (≥16 mm), the melanoma-related mortality at 25 years was 18%, 52%, and 59%, respectively [9]. In a research of 8,033 UM patients by Shields et al. [10], there was a 5% increased risk for metastasis at 10 years in the condition that tumor thickness increased by one millimeter. The female gender was significantly associated with a lower risk of disease-specific mortality than the male gender by survival analysis of 119 patients [11]. Similarly, a study of 723 uveal melanoma patients found that males had an unfavorable prognosis compared with females [12]. In our study, UMPS was more accurate than single clinicopathological factors or the combined clinical model, which is relatively complex and time-consuming to use in an everyday clinical practice setting.

Our results highlight the notion of using genomic disease stratification algorisms for prognosis predicting of UM patients. Similarly, while some authors have tried to use genetic tests to improve the prognostic value of clinicopathological prognostic predictors, other investigators provide the formulas of combined clinical and genetic parameters. For example, Jorge Vaquero et al. developed a web-based prognosis prediction tools PriMeUM [13]. The accuracy of the risk prediction was 80%, 83%, and 85% by prognostic prediction models using chromosomal features only, clinical features only (age, sex, tumor location, and size), and combined clinical and chromosomal information [13]. The web-based tool LUMPO, providing a personalized estimation of survival in UM patients by the combination of pathological, clinical, and genetic data, was developed and externally validated [3]. Ni [14] built a 14 gene expression-based signature derived from the TCGA dataset to predict OS/RFS of UM by using WGCNA and Cox regression analyses, the gene expression classifier had the best AUC compared to clinicopathological features or chromosome aberrations. Luo et al. [15] identified 21 microenvironment-related prognosis genes by using the TCGA cohort as the training cohort and GSE22138 as the validation cohort. Xue et al. [16] performed univariate Cox regression analysis and glmnet Cox analysis on the TCGA cohort to generate the 18-gene prognostic model for patients' OS. Choi et al. [17] performed the log-rank test and univariate Cox regression using GSE22138, GSE39717, and TCGA cohorts. A total of 37 oncogene-like and 14 tumor suppressor-like genes were intersected among the three cohorts. Protein-protein analysis revealed NDUFB9, NDUFV2, CYC1, and CTNNB1 may be prognostic molecular predictors in UM. Luo et al. [18] performed Kaplan–Meier analysis, univariate Cox regression, and Lasso-Cox to build a ten-gene signature using TCGA cohort and validated the signature using GSE22138 cohort. Our UMPS for the GSE22138 cohort was more accurate in predicting 3-y and 5-y MFS (AUC = 0.794 and 0.867, respectively), compared to Luo et al. (AUC = 0.785). Our study differed from the abovementioned ones. First, we performed a combination of WGCNA and Lasso-Cox regression analysis. Second, we derived the model from the GSE22138 cohort and validated with the TCGA cohort. Third, we mainly focused on building a genomic model to predict metastasis of UM. We provided a possible new genomic predictive system to evaluate metastasis risk for patients with UM by evaluating the gene expression in the local UM samples. Whether using UMPS alone or in combination with clinicopathological factors warrant further investigation in different clinical centers or via prospectively designed study.

Among the eight genes in UMPS, EIF1B has been investigated previously in UM, and the other seven were found novel to UM. Harbour and coworkers described a 12 genes expression profile predictive of systemic metastasis in uveal melanoma including EIF1B [19]. Different from our findings of RPL10A as a protective prognostic marker, low-expression of RPL10A was associated with worse OS and RFS of patients with glioblastoma [3] and worse OS of patients with breast cancer [20]. TIPARP act as a tumor suppressor in breast cancer that might be regulated by DNA methylation [21]. Lower TIPARP expression was related to unfavorable survival, and the expression of TIPARP was upregulated by metformin treatment [21]. On the contrary, TIPARP was upregulated in meningioma [22]. While RPL15 was downregulated in cutaneous squamous cell carcinoma [23] and pancreatic ductal adenocarcinoma [3], it was markedly upregulated in gastric cancer [3] and colon cancer tissues [24]. SLC25A38 protein level was higher in ALL patients compared to controls. But, decreased expression of SLC25A38 was found in hepatocellular carcinoma and associated with microvascular invasion [25]. PHLDA1 was downregulated and could act as a protective prognosis biomarker in hepatocellular carcinoma [26]. In addition, reduced expression of PHLDA1 could enhance proliferate and migration of breast cancer cells and was related to unfavorable prognosis [27]. TFDP2 was decreased in human papillary carcinoma tissue, and its expression can be altered by CDDP treatment [28]. EGF10 was identified to contribute to tumorigenesis of medulloblastoma from in vitro and in vivo experiments [3]. As indicated by GSEA, high-risk group showed various activated cancer pathways. The E2F1 targets pathway was involved in the BAP1-mediated cell cycle progression of uveal melanoma cells [3]. The G2M checkpoint pathway was a key element of cell cycle regulation function. A higher score of the G2M checkpoint pathway was an independent negative prognostic factor of pancreatic cancer [3]. Growing evidence suggests that the tumor immune microenvironment contributes to cancer progression including uveal melanoma [3]. However, our result did not find a difference in immune cells between the high-risk and low-risk groups as defined by the UMPS formula. Although immune cell infiltration in the tumor microenvironment contributes significantly to UM prognosis, it does not contribute to the worse prognosis of the high-UMPS patients. Instead, high-UMPS patients' worse prognosis may be due to the activated well-known oncogenic pathways, such as E2F targets, G2M checkpoint, and unfolded protein response pathways. These results may implicate that our formula predicts disease progression of UM independent of the tumor microenvironment.

Taken together, further characterization of the eight genes will give new insights into the understanding of UM development and disease progression and may contribute to the discovering of potential therapeutic targets for patients with UM.

The current study had several limitations. First, the sample size of the development and validation cohorts is relatively small. Second, the biological function and mechanism of the identified eight genes in UM are still unknown. Third, this predicting formula was developed from UM microarray data from France and validated in the TCGA data from the United States, whether the UMPS formula could be implemented in different populations requires further study.

## 5. Conclusion

In conclusion, the 8-gene expression-based UMPS formula is a promising prognostic tool for predicting metastasis and survival for patients with UM. The additional UMPS may augment the predicting accuracy of traditional clinicopathological predictors. Using UMPS, high-risk patients could be subjected to additional treatment such as adjuvant chemotherapy. Conversely, low-risk patients can be spared to unnecessary surveillance and treatment to prevent tumor metastasis.

## Data Availability

All data could be available from https://www.ncbi.nlm.nih.gov/geo/ and https://xenabrowser.net/.

## Supplementary Materials

Supplementary Materials Supplementary Figure 1: the discriminative power of UMPS for disease-specific survival (a) and overall survival (b) in the TCGA cohort. Supplementary Figure 2: validation of UMPS in the GSE27831 cohort. (a) Risk score distribution. (b) Survival overview. (c) Survival curve of UMPS for disease-free survival (DFS). The high-risk group had worse DFS than the low-risk group. (d, e) Time-dependent ROC analysis for comparing the performance of UMPS with the clinicopathological factors and the other genomic factors. The AUC of UMPS was higher than the single clinicopathological factor in predicting 3-y and 5-y DFS. Supplementary Table 1: univariate Cox regression analysis of metastasis-free survival in the training cohort. Supplementary Table 2: multivariate Cox regression analysis of metastasis-free survival in the training cohort. Supplementary Table 3: univariate Cox regression analysis of metastasis-free survival in the validation cohort. Supplementary Table 4: univariate Cox regression analysis of disease-specific survival in the validation cohort. Supplementary Table 5: multivariate Cox regression analysis of disease-specific survival in the validation cohort. Supplementary Table 6: univariate Cox regression analysis of overall survival in the validation cohort. Supplementary Table 7: multivariate Cox regression analysis of overall survival in the validation cohort.
