# Supplementary material for: A Novel 8-Gene Prognostic Signature for Survival Prediction of Uveal Melanoma
Source: Anal Cell Pathol (Amst). 2021 Aug 14;2021:6693219. doi: 10.1155/2021/6693219 (PMC8382551; doi:10.1155/2021/6693219)
Supplement: Supplementary Materials — Supplementary Figure 1: the discriminative power of UMPS for disease-specific survival (a) and overall survival (b) in the TCGA cohort. Supplementary Figure 2: validation of UMPS in the GSE27831 cohort. (a) Risk score distribution. (b) Survival overview. (c) Survival curve of UMPS for disease-free survival (DFS). The high-risk group had worse DFS than the low-risk group. (d, e) Time-dependent ROC analysis for comparing the performance of UMPS with the clinicopathological factors and the other genomic factors. The AUC of UMPS was higher than the single clinicopathological factor in predicting 3-y and 5-y DFS. Supplementary Table 1: univariate Cox regression analysis of metastasis-free survival in the training cohort. Supplementary Table 2: multivariate Cox regression analysis of metastasis-free survival in the training cohort. Supplementary Table 3: univariate Cox regression analysis of metastasis-free survival in the validation cohort. Supplementary Table 4: univariate Cox regression analysis of disease-specific survival in the validation cohort. Supplementary Table 5: multivariate Cox regression analysis of disease-specific survival in the validation cohort. Supplementary Table 6: univariate Cox regression analysis of overall survival in the validation cohort. Supplementary Table 7: multivariate Cox regression analysis of overall survival in the validation cohort. [file 6693219.f1.doc]

## A novel 8-gene prognostic signature for survival prediction of uveal melanoma

Zhongjun Tang^1^, Kebo Cai^2*^

1. Department of Ophthalmology, Minhang Hospital, Fudan University, Shanghai, China, [tangzhongjun1988@163.com](mailto:tangzhongjun1988@163.com)
2. Department of Ophthalmology, Xinhua Hospital Affiliated to Shanghai Jiao Tong University School of Medicine, Shanghai, China, caikebo2019@126.com

^*^Correspondence: Kebo Cai, Department of Ophthalmology, Xinhua Hospital Affiliated to Shanghai Jiao Tong University School of Medicine, Shanghai, China, caikebo2019@126.com

**Supplementary Fig. 1** The discriminative power of UMPS for disease specific survival (A) and overall survival (B) in the TCGA cohort.

**Supplementary Fig. 1**

**
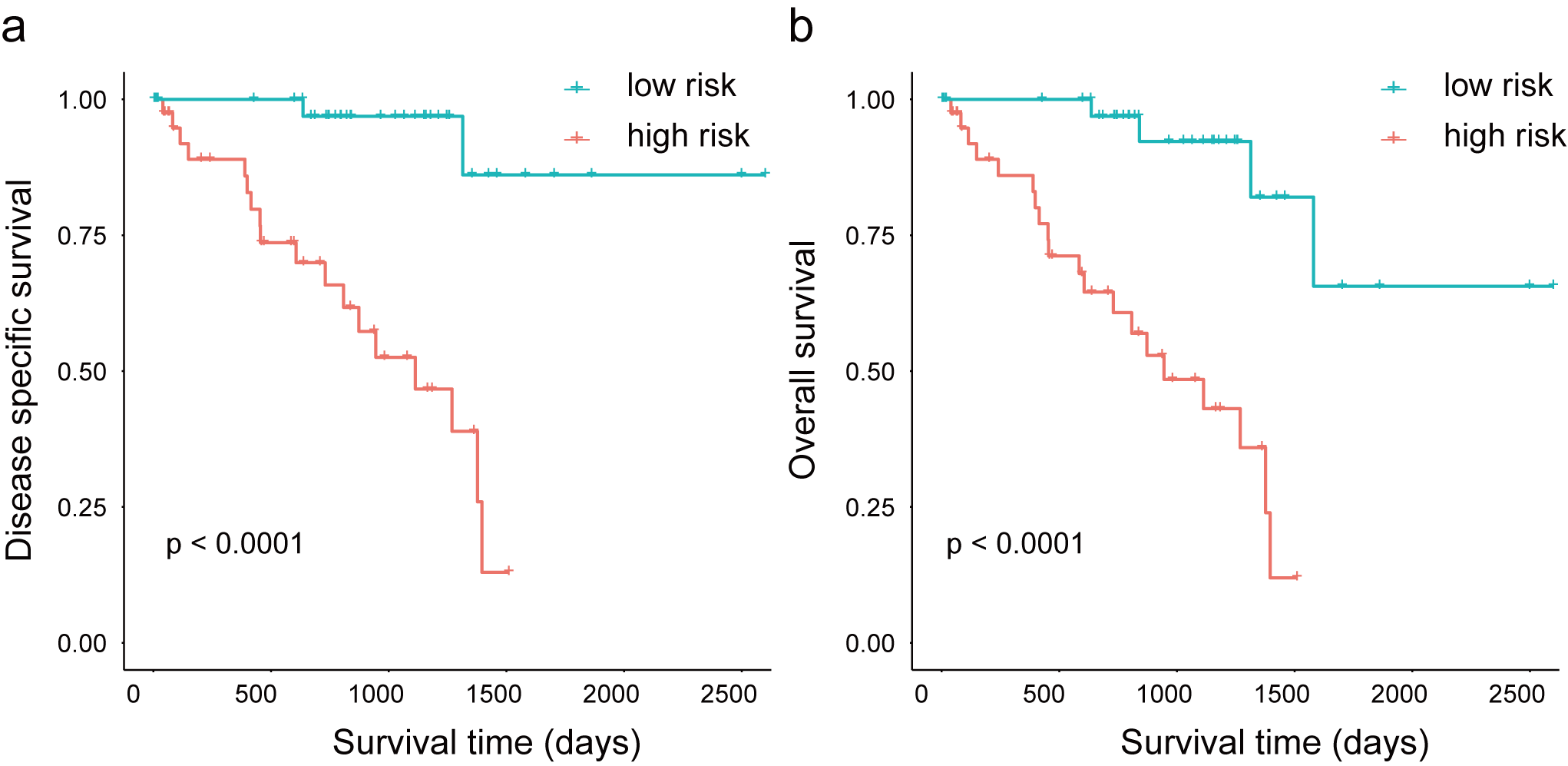
**

**Supplementary Fig. 2** Validation of UMPS in the GSE27831 cohort. a. Risk score distribution；b. Survival overview; c. Survival curve of UMPS for disease free survival (DFS). High-risk group had worse DFS than low-risk group; d-e. Time-dependent ROC analysis for comparing performance of UMPS with the clinicopathologcial factors and the other genomic factors. The AUC of UMPS was higher than single clinicopathological factor in predicting 3-y and 5-y DFS.

**Supplementary Fig. 2**


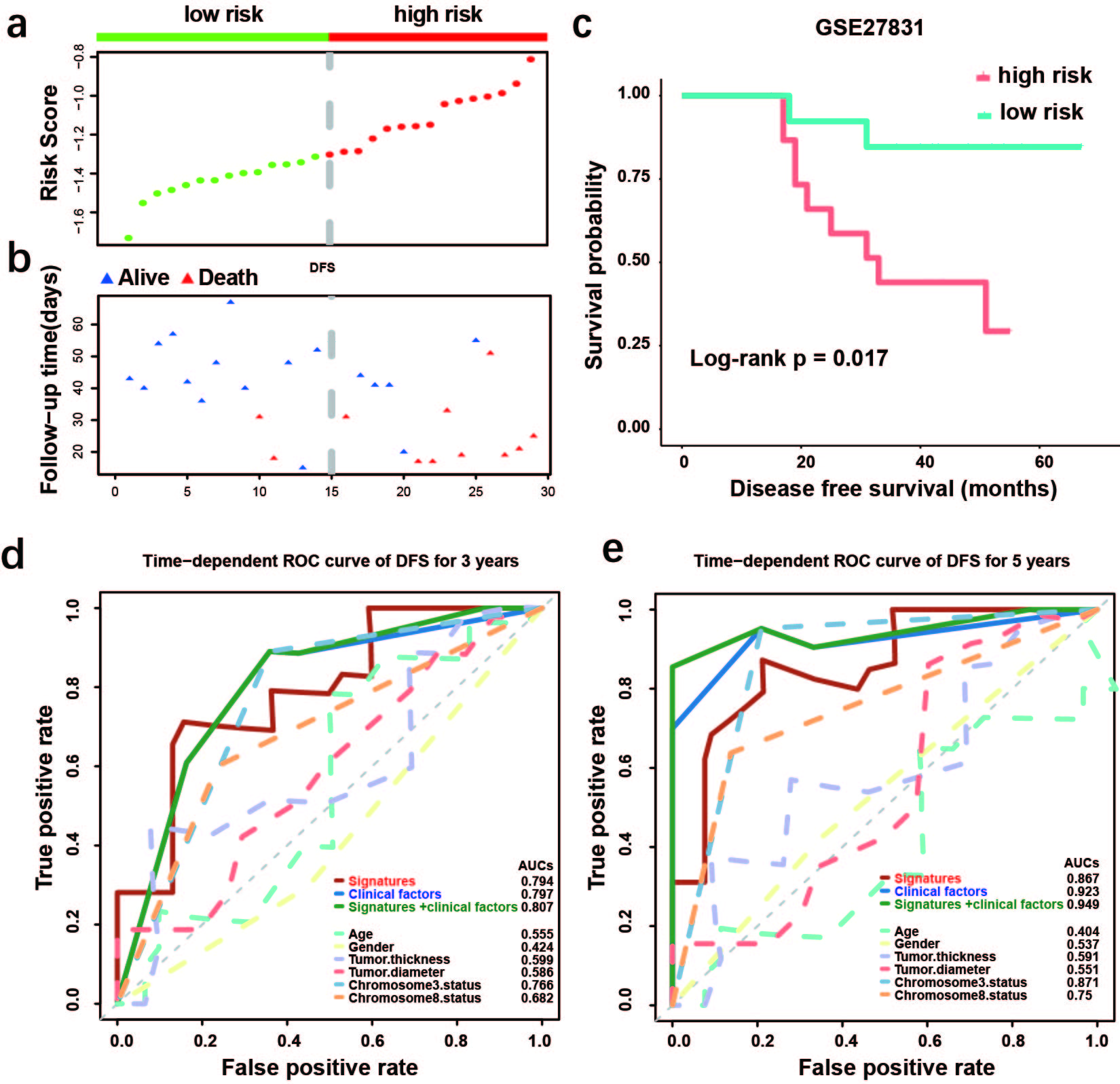


**Supplementary Table 1. Univariate Cox regression analysis of metastasis free survival in the training cohort**

|  | beta | HR | (95%CI for HR) | score.test | p.value |
| --- | --- | --- | --- | --- | --- |
| Age(continuous) | 0.02 | 1 | (0.99-1) | 1.9 | 0.17 |
| **Chromosome3.status**  (monosomy vs partial monosomy/disomy) | 1.4 | 4 | (1.7-9.8) | 11 | **0.00099** |
| Eye(right vs left ) | 0.18 | 1.2 | (0.55-2.6) | 0.21 | 0.64 |
| Gender(female vs male) | 0.0079 | 1 | (0.46-2.2) | 0.00039 | 0.98 |
| Retinal.detachment(yes vs no) | 0.77 | 2.2 | (0.91-5.1) | 3.2 | 0.074 |
| Tumor.thickness(continuous) | 0.15 | 1.2 | (0.94-1.4) | 2 | 0.16 |
| **Sigantures** (high-risk vs low-risk) | 1.8 | 6.2 | (2.5-15) | 20 | **8.60E-06** |

**Supplementary Table 2. Multivariate Cox regression analysis of metastasis free survival in the training cohort**

|  | coef | exp(coef) | se(coef) | z | p |
| --- | --- | --- | --- | --- | --- |
| Chromosome3.status  (monosomy vs partial monosomy/disomy) | 0.02336 | 1.02364 | 0.71307 | 0.033 | 0.9739 |
| **Sigantures**(high-risk vs low-risk) | 1.80721 | 6.09345 | 0.7201 | 2.51 | **0.0121** |

**Supplementary Table 3. Univariate Cox regression analysis of metastasis free survival in the validation cohort**

|  | beta | HR | (95%CI for HR) | score.test | p.value |
| --- | --- | --- | --- | --- | --- |
| Age(continuous) | 0.016 | 1 | (0.98-1) | 0.96 | 0.33 |
| Gender(female vs male) | -0.25 | 0.78 | (0.34-1.8) | 0.35 | 0.55 |
| Stage(>stageⅡ vs ≤stageⅡ) | 0.87 | 2.4 | (0.94-6) | 3.6 | 0.059 |
| Tumor.diameter(continuous) | 0.13 | 1.1 | (0.97-1.3) | 2.4 | 0.12 |
| Connective.loop(present vs absent) | 0.68 | 2 | (0.86-4.5) | 2.7 | 0.1 |
| Pigmentation(moderate/heavy vs mild) | 0.55 | 1.7 | (0.75-4) | 1.7 | 0.19 |
| Tumor.thickness(continuous) | 0.12 | 1.1 | (0.94-1.3) | 1.7 | 0.2 |
| Sigantures(high-risk vs low-risk) | 2.1 | 8.5 | (2.8-25) | 20 | **6.20E-06** |

**Supplementary Table 4. Univariate Cox regression analysis of disease specific survival in the validation cohort**

|  | beta | HR | (95%CI for HR) | score.test | p.value |
| --- | --- | --- | --- | --- | --- |
| Age(continuous) | 0.042 | 1 | (1-1.1) | 4.4 | **0.036** |
| Gender(female vs male) | -0.63 | 0.53 | (0.2-1.4) | 1.7 | 0.2 |
| Stage(>stageⅡ vs ≤stageⅡ) | 0.66 | 1.9 | (0.69-5.4) | 1.7 | 0.2 |
| Tumor.diameter(continuous) | 0.13 | 1.1 | (0.94-1.4) | 1.6 | 0.2 |
| Connective.loop(present vs absent) | 0.43 | 1.5 | (0.62-3.8) | 0.88 | 0.35 |
| Pigmentation(moderate/heavy vs mild) | 0.99 | 2.7 | (1-7.1) | 4.1 | **0.042** |
| Tumor.thickness(continuous) | 0.21 | 1.2 | (0.99-1.5) | 3.6 | 0.057 |
| Sigantures(high-risk vs low-risk) | 2.6 | 13 | (3-57) | 19 | **1.10E-05** |

**Supplementary Table 5. Multivariate Cox regression analysis of disease specific survival in the validation cohort**

|  | coef | exp(coef) | se(coef) | z | p |
| --- | --- | --- | --- | --- | --- |
| Age(continuous) | 0.06124 | 1.06316 | 0.0262 | 2.337 | **0**.**019427** |
| Pigmentation(moderate/heavy vs mild) | 0.4052 | 1.49961 | 0.57441 | 0.705 | 0.480548 |
| Sigantures(high-risk vs low-risk) | 2.79479 | 16.35916 | 0.79875 | 3.499 | **0.000467** |

**Supplementary Table 6. Univariate Cox regression analysis of overall survival in the validation cohort**

|  | beta | HR | (95%CI for HR) | score.test | p.value |
| --- | --- | --- | --- | --- | --- |
| Age(continuous) | 0.057 | 1.1 | (1-1.1) | 8.7 | **0.0032** |
| Gender(female vs male) | -0.46 | 0.63 | (0.27-1.5) | 1.1 | 0.29 |
| Stage(>stageⅡ vs ≤stageⅡ) | 0.5 | 1.6 | (0.67-4) | 1.2 | 0.27 |
| Tumor.diameter(continuous) | 0.18 | 1.2 | (1-1.4) | 4.1 | **0.042** |
| Connective.loop(present vs absent) | 0.39 | 1.5 | (0.65-3.4) | 0.87 | 0.35 |
| Pigmentation(moderate/heavy vs mild) | 1.1 | 3 | (1.2-7.3) | 6.1 | **0.014** |
| Tumor.thickness(continuous) | 0.15 | 1.2 | (0.96-1.4) | 2.5 | 0.11 |
| Sigantures(high-risk vs low-risk) | 2.3 | 9.6 | (2.8-33) | 19 | **1.10E-05** |

**Supplementary Table 7. Multivariate Cox regression analysis of overall survival in the validation cohort**

|  | coef | exp(coef) | se(coef) | z | p |
| --- | --- | --- | --- | --- | --- |
| Age(continuous) | 0.08073 | 1.08408 | 0.02466 | 3.274 | **0.00106** |
| Tumor.diameter(continuous) | 0.14748 | 1.15891 | 0.12195 | 1.209 | 0.226543 |
| Pigmentation(moderate/heavy vs mild) | 0.39287 | 1.48122 | 0.55269 | 0.711 | 0.477189 |
| Sigantures(high-risk vs low-risk) | 2.40583 | 11.08763 | 0.67121 | 3.584 | **0.000338** |
